# Draft genome sequence of *Bacillus velezensis* strains AOA1 and AKS2, the potential plant growth-promoting rhizobacteria

**DOI:** 10.1128/mra.00877-23

**Published:** 2024-02-27

**Authors:** Olubukola Oluranti Babalola, Akinlolu Olalekan Akanmu, Ayansina Segun Ayangbenro

**Affiliations:** 1 Food Security and Safety Focus Area, Faculty of Natural and Agricultural Sciences, North-West University, Mafikeng, South Africa; California State University San Marcos, USA

**Keywords:** bacteria, PGPR, Illumina Nextera, genome assembly, DNA extraction, KBase, soil microbe extraction

## Abstract

This report describes the draft genome sequence of *Bacillus velezensis* strains AOA1 and AKS2 isolated from maize rhizosphere soil in South Africa. *Bacillus velezensis* plays important biological roles as plant growth promoting rhizobacterium (PGPR). *Bacillus velezensis* strains also exhibit numerous biotechnological application potentials in agriculture and diverse industrial settings.

## ANNOUNCEMENT

*Bacillus* species are endospore-forming and rod-shaped bacteria, which are either aerobic or facultative anaerobe. Many species exhibit a wide range of physiological traits, which enable their survival in a diverse natural environment (1). *Bacillus velezensis* was formerly classified as strains of *Bacillus amyloliquefaciens* but reclassified based on genomic analysis (2, 3). Comparative genomics and DNA-DNA relatedness calculations had, however, showed that *B. velezensis* was taxonomically distinct from *B. amyloliquefaciens* (4). Many of the *B. velezensis* strains are gaining relevance for their applications in medicine, pharmaceutics, food industry, and environment (5). Moreover, this species is harmless to both humans and animals, and environmentally safe (4).

Experimental samples were collected at the North-West University Research Farm, Molelwane, South Africa (25°47′26.4″ S 25°37′01.6″ E) on March 2022. Maize plants were randomly selected, uprooted, and 10 g of soil tightly adhering to maize roots was scraped. Isolation of bacteria from the soil samples was carried out by serial dilution, plated on Luria-Bertani (LB) agar plates and incubated at 28°C ± 2°C for 24 h. Individual bacterial colonies were picked and streaked on LB plates for further purification. The growth from a single colony was selected and confirmed through morphological and biochemical tests in-line with the protocol described by Majeed et al. (6).

A ZF Fungal/Bacterial DNA MiniPrep Extraction Kit by Zymo Research, USA was used for genomic DNA extraction from pure culture grown on solid agar. The genome of each strain was subsequently sequenced at Novogene Co. Ltd., Singapore. Library was prepared from the DNA sample using Illumina Nextera DNA Flex Library Preparation Kit. The prepared library was sequenced using NovaSeq 6000 (2 × 150 bp).

The whole-genome sequencing analysis was conducted with KBase (7). FastQC v43.1.0.4 (8) was employed in examining the read quality, while adapter regions and low-quality reads were filtered using Trimmomatic v1.2.14 (9). The total read pairs of 4,394,405 and 3,455,403 were obtained after filtering and trimming for *Bacillus velezensis* strains AOA1 and AKS2, respectively. The genome assembly was performed using SPAdes v1.2.4 (10). The assembled genomes were subjected to BLAST assessment using GTDB-Tk v1.7.0 (11), which showed FastANI taxonomy of 98.13 and 98.08 with *Bacillus velezensis,* respectively. The presence of secondary metabolites, such as iturins, fengycins, and surfactins, was revealed in both strains with antiSMASH (version 7.0.1) (12). The annotation for prediction of gene functions was conducted using the NCBI Prokaryotic Genome Annotation Pipeline (13) (Table 1).

**TABLE 1:** Whole-genome sequencing characteristics of *Bacillus velezensis* strains AOA1 and AKS2

Parameter	*Bacillus velezensis*	
	Strain AOA1	Strain AKS2
Genome size	3,919,636	3,935,018
G + C contents (%) (bp)	46.43	46.38
Number of contigs	12	21
Total read pairs	4,394,405	3,455,403
N50 (bp)	2,072,719	1,238,655
L50	1	2
FastANI taxonomy	98.13	98.08
Number of genes (total)	3,873	3,888
DNA sequences (CDSs)	3,820	3,734
Number of proteins	3,746	3,734
Number of rRNAs	1, 2, 1 (5S, 16S, 23S)	2, 3, 2 (5S, 16S, 23S)
Number of tRNAs	44	56
Number of pseudogenes	74	86
Noncoding sequences	53	68
Noncoding RNAs	5	5

## Data Availability

The whole-genome sequences of *Bacillus velezensis* strains AOA1 and AKS2 have been deposited at DDBJ/ENA/GenBank under the accession numbers JAVHYM000000000 and JAVKYN000000000. The versions described in this paper are versions JAVHYM000000000.1 and JAVKYN000000000.1, respectively. Also, the raw reads are available under BioProject accession numbers PRJNA1010530 and PRJNA1014219, with the BioSample accession numbers SAMN37190764 and SAMN37321166. The sequence data obtained in this work have been deposited in the NCBI. Sequence Read Archive under the accession numbers SRR25784552 and SRR25949163 for strains AOA1 and AKS2, respectively.
